# Robotic Assisted TKA achieves adjusted mechanical alignment targets more consistently compared to manual TKA without improving outcomes

**DOI:** 10.1002/jeo2.70008

**Published:** 2024-09-02

**Authors:** Ashok Rajgopal, Sriram Surendra Sundararajan, Kalpana Aggarwal, Sumit Kumar, Gargi Singh

**Affiliations:** ^1^ Institute of Musculoskeletal Disorders and Orthopaedics Medanta‐The Medicity Hospital Gurugram India; ^2^ Institute of Education and Research Medanta‐The Medicity Hospital Gurugram India

**Keywords:** conventional total knee arthroplasty, gait analysis, patient reported outcome measures, range of motion, robotic total knee arthroplasty

## Abstract

**Introduction:**

Robotic total knee arthroplasty (rTKA), with its purported advantages of more accurate alignment, greater functional outcomes and patient satisfaction, is gaining popularity in patients undergoing TKA. The purpose of our study was to compare these parameters along with gait pattern and kneeling ability in a cohort of patients who underwent simultaneous TKA with manual instrumentation (mTKA) and rTKA in contralateral knees at a 1‐year follow‐up.

**Methods:**

This was a retrospective review of 135 consecutive patients who underwent simultaneous bilateral TKA using robotic assistance on one side and manual instrumentation on the contralateral side between January 2022 and June 2022. The target alignment in both cohorts was adjusted mechanical. Patients were followed up at 3, 6 and 12 months to assess and compare alignment, range of motion (ROM) and patient‐reported outcome measures (PROM) data. Gait parameters and kneeling ability were assessed at 1‐year follow‐up.

**Results:**

While adjusted mechanical alignment was achieved in all rTKA patients, we recorded five outliers (≥3° with relation to 180° HKA axis) in the mTKA cohort (three varus and two valgus). There were no significant differences between both cohorts with regards to ROM, PROM scores, gait analysis parameters and kneeling ability at 1‐year follow‐up.

**Conclusion:**

rTKA helps in achieving the adjusted mechanical alignment more consistently than mTKA. This, however, does not contribute to better functional outcomes and patient satisfaction at 1‐year follow‐up.

**Level of Evidence:**

Level III.

**Abbreviations**
HSS: hospital for special surgery
KSS: knee function scores
mTKA: TKA with manual instrumentation
OKS: Oxford knee score
PROM: patient‐reported outcome measures
ROM: range of motion
ROSA: robotic surgical assistant
rTKA: robotic total knee arthroplasty
TKA: total knee arthroplasty
WOMAC: Western Ontario and McMaster University Osteoarthritis index

## INTRODUCTION

TKA is the treatment of choice for end‐stage knee arthritis, with recent studies projecting an increase of 139% by 2040 and 469% by 2060 [33]. Despite improved patient satisfaction rates over the years, about 5%–20% of the patients continue to be dissatisfied [8, 37]. Various authors have reported that restoration of alignment and soft tissue balancing will lead to improved patient satisfaction and outcomes [13, 29, 31]. Surgeon‐controlled variables that impact outcomes include restoration of a pre‐planned alignment, balanced soft tissue tension and flexion‐extension gap [7]. Enabling technology such as rTKA has been developed with the objective of achieving these goals in terms of accuracy and alignment to help improve outcomes. rTKA has evolved over the last decade with increasing evidence of improved component alignment as compared to mTKA [4, 23, 24, 36]. The advantages of rTKA are the surgeons' ability to target different alignment options such as unrestricted kinematic alignment, reverse kinematic alignment and functional alignment. Robotic intervention allows for achieving these targets with predictability and accuracy.

The first rTKA was performed in 1988 using ACROBOT robotic system in the United Kingdom [32]. There have been significant developments since and a number of robotic devices are available today. Robotic systems have been classified into semi‐active and fully active systems depending on the surgeon's external control.

There are differences between image‐based and imageless plans: The 3D bone model, axial view and the option buttons for presenting the landmarks, axis, cuts and implants are available only with image‐based plans. This helps in creating a virtual plan for implant positioning and ligament balancing thereby aiding the surgeon in manually performing bone resections guided by robotic‐positioned cutting blocks [2, 20]. ROSA is a closed platform semi‐active robotic (image‐based) system which converts two‐dimensional knee radiographs into a 3D patient‐specific bone model [9].

This study was undertaken to evaluate

(a) Consistency in achieving adjusted mechanical alignment in the manual and robotic cohorts.
(b) Functional outcomes and patient satisfaction.
(c) Gait parameters and kneeling ability in patients undergoing simultaneous rTKA and mTKA in contralateral knees, at 1‐year follow‐up.

The purpose of this study was to evaluate if targeting adjusted mechanical alignment using manual intervention and robotic technology on contralateral knees altered outcomes, results and patient satisfaction. We did not use differential alignment options in each knee to avoid introducing variables and biases in the final evaluation of outcomes. As this was a single‐stage bilateral TKA with manual and robotic options on contralateral knees, each patient served as their own control.

The null hypothesis of this study was that using robotic assistance would improve alignment, outcomes and PROMs and reduce the incidence of dissatisfaction in this cohort.

## MATERIALS AND METHODS

### Patient selection

This was a retrospective single‐blinded study undertaken in accordance with the Helsinki Declaration and duly approved by the Institutional Review Board (IRB registration number 1691/2024). A cohort of 135 consecutive patients who underwent simultaneous bilateral TKA for end‐stage arthritis between January 2022 and June 2022 was selected for this study. Inclusion criteria for this study included patients with bilateral varus deformity of degenerative aetiology.

Exclusion criteria for this study included patients with windswept deformities, valgus deformities, fixed flexion and hyperextension deformities, arthritis of inflammatory aetiology, posttraumatic arthritis and those with grade 3 ligament injuries. In this study, 25% of the patients were CPAK Type I, 23% were CPAK Type II and 44% were CPAK Type IV. CPAK Type III, V and VII accounted for the remaining 8%. No patellar resurfacing was done in any patient in this study.

A slip selection method was used [34], for robotic intervention (ROSA, Zimmer‐ Biomet, Inc.) on one side and conventional manual instrumentation on the other. All patients were blinded to this variable.

### Surgical technique

All patients were operated by the senior surgeon. All of them received a spinal block. A pneumatic tourniquet was used and set at 280 mm Hg. Arthrotomy was performed using the standard medial parapatellar approach. The chosen targeted alignment was mechanical with the aim of restoring the HKA angle of 180° (177°–183° safe HKA). For the mTKA cohort, an intramedullary guide rod was placed in the femur and distal femoral cut was taken at 3° valgus. The distal femur cutting block was then placed in 3° of external rotation in reference to the posterior condylar axis following which anterior femur, posterior condyles and chamfer cuts were executed. For the tibial resection, an extramedullary cutting jig was used and the surgeon aimed for a neutral tibial cut. Mediolateral flexion and extension gaps were checked with trial components.

In rTKA, the system was optimally positioned, followed by an identical arthrotomy and optical reflective trackers were inserted from within the wound margins. Subsequently, a dynamic knee evaluation was carried out to record ROM, alignment and joint laxity. A surgical plan was mapped out and robotic arm‐assisted bone cuts were executed based on native anatomy and inputs from the software. All resected bone specimens were measured with a calliper to corroborate the accuracy of the resection. All cases were operated using an imageless ROSA system.

Components were implanted after pulsed lavage irrigation, drying and pressurisation of cement. A medial congruent design retaining the posterior cruciate ligament was used in all knees. Optimum polyethylene thickness was chosen to neutralise joint laxity and restore stable ROM. None of the patellae were resurfaced. Prior to closure, all knees were infiltrated with a multi‐site periarticular uniform preparation of an analgesic cocktail. All patients received an ultrasound‐guided adductor canal block without disturbing the sterile dressing. All patients in this study received Persona® (Zimmer Inc.) implants with identical pre‐ and peri‐operative protocols.

The drain was removed 24 h after surgery during a dressing check. Prophylactic antibiotics were administered for 2 days. All patients in the study received DVT prophylaxis in the form of mechanical compression using DVT pumps for the first four postoperative days in addition to injection Enoxiparin 60 mg (subcutaneous) for 2 weeks followed by 6 weeks of Aspirin therapy (150 mg OD).

All patients were mobilised with a walker 4 h after surgery after assessing their ability to achieve active SLR. They were trained to ambulate on flat ground with an assistive device and were discharged with instructions to perform and complete a set of exercises at home.

### Clinical evaluation

The primary outcomes of interest were post‐operative radiographic parameters including HKA angle and number of radiographic outliers, and differences in PROMs between the two cohorts. Secondary outcomes of interest were gait analysis parameters and kneeling ability between the two groups.

Early data points checked included pain scores, time and ability to perform straight leg raises (SLR), time taken to achieve 90° of flexion and to climb stairs. Late data points included evaluating component alignment, kneeling ability, gait parameters including forefoot, midfoot and heel pressure and PROM data. The main outcome measures were the PROM data (OKS and KSS) [10], which were evaluated at 3 months, 6 months and one year.

ROM was measured by a senior physiotherapist using a long‐arm handheld goniometer.

Gait analysis of all patients was performed at 1‐year follow‐up [14]. Plantar pressure namely forefoot, midfoot and heel pressures, in Newtons/cm^2^, was measured and recorded using a Zebris™ FDM‐2 (zebris® Medical GmbH) device. All patients walked individually without any assistance and were asked to take at least 10 steps on the walking platform before the analysis began so that their natural gait pattern could be recorded.

Kneeling ability after TKA was assessed at 1‐year follow‐up. For assessing kneeling ability, patients were asked to demonstrate how comfortable they were with kneeling on hard, moderate and soft surfaces and their responses were documented.

Radiographs including standing knee AP and lateral radiographs were recorded preoperatively, immediately postoperatively and at 1‐year follow‐up. An orthoscanogram and a merchant view were done preoperatively and at 3‐month follow‐up. All data were recorded by a musculoskeletal radiologist who was not a part of the operating team.

### Statistical analysis

Descriptive analysis of quantitative parameters was expressed as means with standard deviation or median and interquartile range. Categorical data were expressed as absolute number and percentage. Cross tables were generated and a chi‐square test was used for testing of associations. Independent Student *t*‐test/Manis study, the sample size is 135 in each of the two groups. n‐Whitney *U* test was used for testing of mean/median between independent groups, Robotic and Manual, whereas repeat measure ANOVA was used for repeat observations at different time points. *p* < 0.05 was considered statistically significant. All analyses were done using SPSS software, version 24.0. In th.

Based on the post hoc power analysis, the power of the present study for comparison of Robotic and Manual Surgery for ROM works out as 50.5% with 95% confidence level. However, for the improvement (Baseline to 12 months) in ROM and KSS for each of the two groups, that is, Robotic and Manual Surgeries, the power is over 90%.

The PROMs were evaluated pre and postoperatively, at each visit, by the same senior physiotherapist. The HKA angles were measured by an independent senior Musculoskeletal Radiologist both, pre‐operatively and at 3 months postoperatively. This was done to eliminate any biases of inter‐observer reliability.

## RESULTS

Of the 135 patients in the study, 104 were females and 31 males. The mean age of patients was 61.1 ± 8.4 years. The mean BMI was 29.8 ± 2.7 kg/m^2^ (Table 1).

**Table 1:** Demographics.

Total number of patients	Mean age of all patients	Mean BMI of all patients
***n*** = **135** **Male 31** **Female 104**	61.1 ± 8.4	29.8 ± 2.7

### Duration of surgery

The time taken for surgery was more in the rTKA cohort as compared to the mTKA cohort. This difference in time was statistically significant (mTKA side 26.1 ± 1.2, rTKA side 29.0 ± 1.4 *p* ≤ 0.001).

### Radiographic measurements

**Figure 1:**
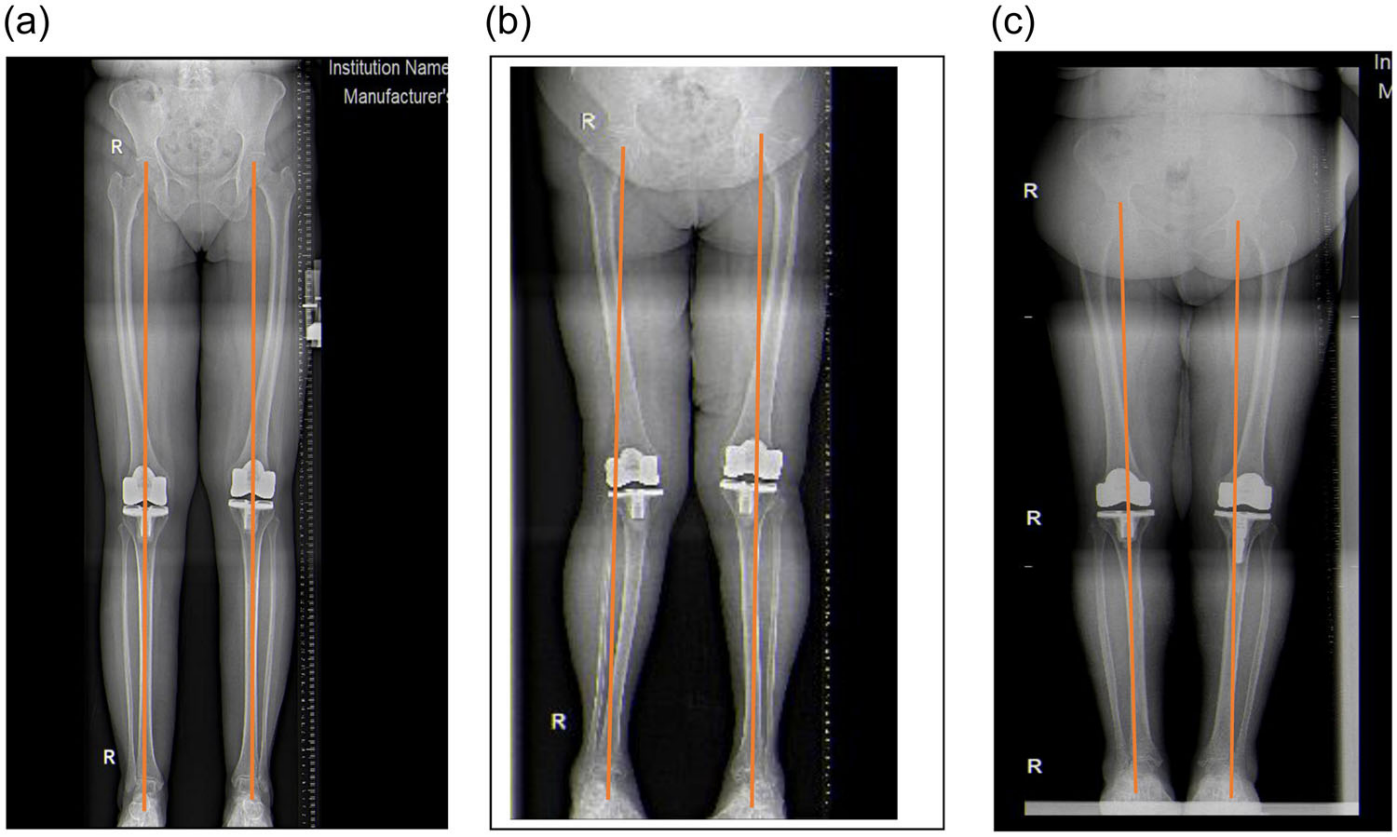
(a) Orthoscanograms of the patient showing both neutrally aligned knee, (b) valgus outlier and (c) varus outlier.

While each knee in the rTKA cohort achieved targeted mechanical alignment the mTKA cohort recorded five outliers. This difference was, however, not statistically significant. (179.8 ± 1.5 in the rTKA cohort vs. 179.8 ± 2.2 in the mTKA cohort, with a mean error of 0.0 ± 0.2, *p* = 1.0). Of the five outliers, we recorded three patients in varus and two in valgus exceeding the safe zone in the mTKA cohort (Figure 1a–c).

Postoperatively, the CPAK changed in both cohorts. In the mTKA cohort, CPAK Type I changed from 28.4% to 29.6%, Type II from 11.7% to 11.4%, Type IV from 56.2 to 54.6% and the others from 3.5% to 4.1%. In the rTKA cohort, CPAK Type I changed from 29.7% to 29.6%, Type II from 12.2% to 11.9%, Type IV from 55.8 to 56.2% and the others from 2.2% to 2.1%.

### ROM and PROM

At the 6‐month follow‐up, patients in the mTKA cohort exhibited significantly better active ROM compared to the rTKA group (111.1 ± 6.9 vs. 107.8 ± 16.4, *p* = 0.032).

**Figure 2:**
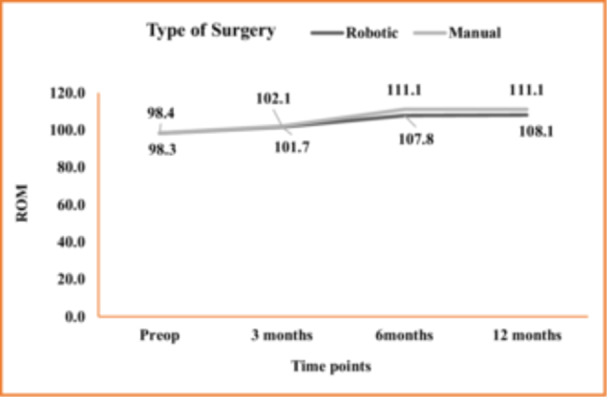
Comparison of the mean value of ROM and KSS at sequential follow‐ups.

At the previous and subsequent follow‐up intervals (i.e., 3 months and 12 months postoperatively), there was no statistically significant difference in active ROM between the two groups (*p* > 0.05) (Figure 2).

During the study period, no patient in the cohort developed deep vein thrombosis, pulmonary embolism, prosthetic joint infection, instability or periprosthetic fractures.

**Figure 3:**
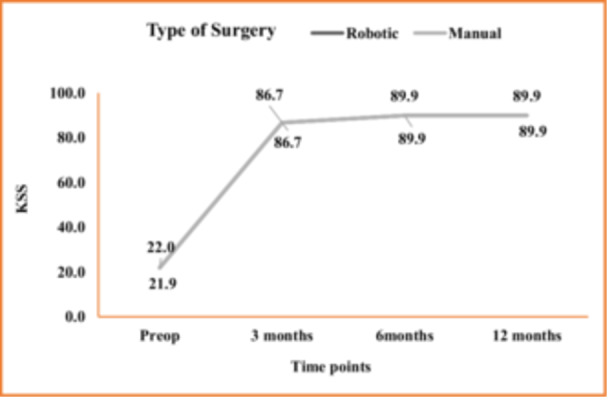
Comparison of the mean value of ROM and KSS at sequential follow‐ups.

We documented KSS scores and OKS scores preoperatively and at every follow‐up until 12 months. Both groups demonstrated a significant increase in scoring postoperatively compared to their preoperative values; however, this increase between the two groups was comparable with no statistical difference (Figure 3). The OKS increased from a preoperative value of 14.3 ± 1.9 to 0.0 ± 0.2 at 12 months postoperatively with a *p* = <0.0001.

### Gait analysis and kneeling ability

**Figure 4:**
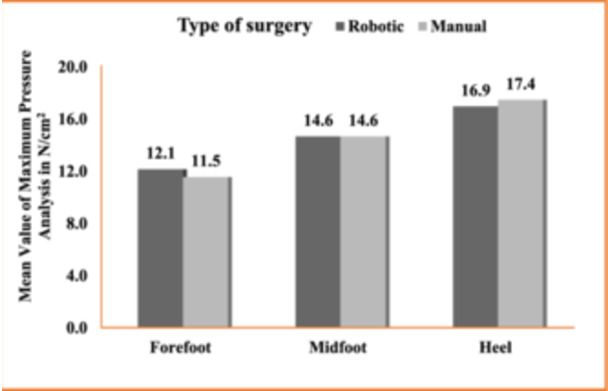
Comparison of the mean value of plantar pressure in gait analysis.

Plantar pressure analysis was recorded at 1 year postoperatively to assess foot loading patterns during walking. At 1‐year follow‐up, it revealed no significant difference in pressure distribution between the mTKA and the rTKA cohort (Figure 4).

**Figure 5:**
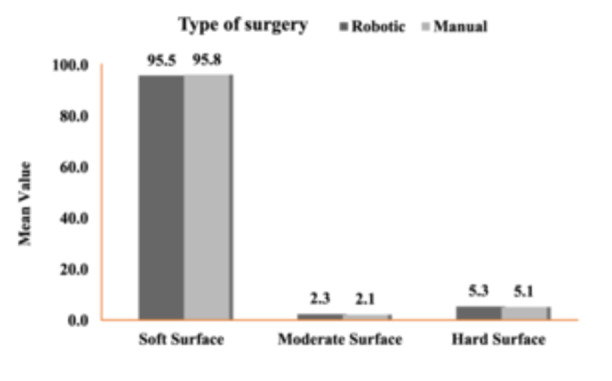
Comparison of the mean value of kneeling ability on different surfaces.

Kneeling ability in both cohorts was tested post‐surgery at 1‐year follow‐up. Patients were asked to kneel over different surfaces: hard (tile/concrete), moderate (carpet) and soft (cushion/pillow). There was no significant difference between the two cohorts in this regard (Figure 5).

## DISCUSSION

The key findings of our study were that despite there being a statistically significant difference in ROM in both cohorts at 6 months postoperatively, there was no difference in PROMs at any stage. Targeted adjusted mechanical alignment was achieved in all knees of the rTKA cohort while the mTKA cohort had five outliers. This difference, however, was not statistically significant (*p* = 1.0) and did not impact outcomes. Similarly, there was no significant difference in gait parameters and kneeling ability between the two cohorts at 1‐year follow‐up.

Mechanical alignment has demonstrated good outcomes and survivorship; however, recent studies have questioned its ability to achieve sound biomechanical targets. Adjusted mechanical alignment was introduced to address this purported shortcoming by adjusting the valgus distal femoral cut while maintaining a 90° tibial resection [3].

Robotic knee systems have demonstrated clear advantages in terms of component alignment which may lead to improved functional outcomes in patients and survivorship of TKA implants. With robotic assistance, surgeons are able to balance each knee individually and achieve the desired objectives with minimum soft tissue damage compared to jig‐based TKA [16, 18, 27, 30, 35]. These advantages, however, stand to be proven in a long‐term follow‐up.

We found no significant differences in OKS and KSS scores postoperatively between the two cohorts in the short term and at 1‐year follow‐up. Yang et al. reported no differences in functional outcomes of WOMAC, HSS and VAS scores between rTKA and mTKA [38]. Similarly, Kim et al. found no differences between navigated and conventional TKA in KSS and WOMAC scores at 15 years of follow‐up [19]. These findings were also similar to studies conducted by Kayani, Bensa and Karunaratne [5, 15, 17].

Song et al. prospectively analysed 50 cases each of rTKA and mTKA and found that the former reduced the probability of inaccurate prosthetic alignment thereby improving the ability to achieve a well‐balanced knee [36]. The present study demonstrated similar findings with all knees mechanically aligned to within the targeted range in the rTKA cohort, while there were five outliers (three varus and two valgus) in the mTKA cohort. This was also similar to Liow et al. who compared rates of mechanical axis deviation and notching and found them to be significantly more in mTKA compared to rTKA [25]. Zhang et al., Agarwal et al. and Kalsan et al. in their studies have reported similar findings [1, 21, 22, 40].

It Is pertinent to emphasise that the robot facilitates the chosen alignment philosophy of the surgeon predictably and repetitively as is seen in our results.

The significantly higher volume of blood loss in the mTKA cohort may be attributed to the fact that the intramedullary canal is breached in mTKA resulting in greater blood loss (413.9 ± 128.4 mL in mTKA as against 206.7 ± 80.9 mL in rTKA, *p* < 0.001).

Gait analysis using quantitative plantar pressure distribution by use of pressure plates records the pressure generated on the plantar surface during weight‐bearing. Degenerative varus knees demonstrate compensatory valgus hindfoot alignment and increased lateral loading rear foot pressure pattern [28]. However, this pattern changes post‐surgery with the restoration of mechanical alignment. We compared the differences in plantar pressures in the forefoot, midfoot and heel during the gait cycle after surgery at 1‐year follow‐up between the two cohorts and found no statistically significant difference between them. Our findings were consistent with the results of the pedobarographic analysis done by Guven et al. and the findings of Cho et al. who recorded change in hindfoot alignment after TKA in varus knees [6, 11].

Several activities of daily living in the Asian and West Asian cultures require acts of kneeling, squatting and sitting cross‐legged. We compared the kneeling ability of patients over different surfaces at 1‐year follow‐up, and all patients from both cohorts were able to kneel over a moderate and a soft surface, more comfortably on the latter. Lynch et al. and others had similar results in their studies with different femoral component designs [26].

This study found that mTKA knees exhibited a significantly greater ROM compared to the rTKA cohort at the 6‐month mark. This discrepancy in outcome between the two surgical approaches is consistent with previous studies that have reported similar findings [12, 39].

Several factors may contribute to this finding. One possible explanation is the variability in surgical technique and soft tissue handling between the two approaches. mTKA typically involves soft tissue releases and ligament balancing often necessitating reduction osteotomies to allow for greater intraoperative flexibility and optimisation of soft tissue tensioning. In contrast, rTKA relies on preoperative planning and robotic guidance for implant placement with limited soft tissue release.

## LIMITATIONS

- This is a short‐term study. A longer follow‐up would be required to address concerns regarding component survivorship, failure rates and the need for revision surgeries.
- The data are of cases operated by a single surgeon and from one institution. Having surgical data from multiple centres may add robustness to this study.

Despite these limitations, this study is valuable as it is a unique comparison of different techniques in a single individual stoking the burning question of whether robotics really makes a worthwhile difference in terms of what is really important from a patient's perspective.

## CONCLUSION

The null hypothesis of our study has not been validated by the outcomes. This study demonstrated that rTKA helps in achieving the adjusted mechanical alignment more consistently than mTKA with significantly less blood loss. This, however, does not contribute to better functional outcomes and patient satisfaction at 1‐year follow‐up. Comparing different alignment strategies in both knees, such as functional alignment may help extend outcome data and add to existing literature. More extensive studies and longer follow‐ups will be necessary to comprehensively compare different aspects of both techniques to arrive at a consensus.

## AUTHOR CONTRIBUTIONS

**Ashok Rajgopal**: Study concept; design; editing. **Sriram Surendra Sundaraajan**: Data curation and writing—original draft. **Sumit Kumar and Kalpana Aggarwal (PT)**: Writing—review and editing. **Gargi Singh**: Analysis of data.

## CONFLICT OF INTEREST STATEMENT

No author had a potential conflict of interest.

## ETHICS STATEMENT

The study was approved by the Medanta Institutional Ethical committee.

## Data Availability

The data sets used and/or analysed during the current study are available from the corresponding author upon reasonable request.
